# Prediction of the COVID-19 Pandemic for the Top 15 Affected Countries: Advanced Autoregressive Integrated Moving Average (ARIMA) Model

**DOI:** 10.2196/19115

**Published:** 2020-05-13

**Authors:** Ram Kumar Singh, Meenu Rani, Akshaya Srikanth Bhagavathula, Ranjit Sah, Alfonso J Rodriguez-Morales, Himangshu Kalita, Chintan Nanda, Shashi Sharma, Yagya Datt Sharma, Ali A Rabaan, Jamal Rahmani, Pavan Kumar

**Affiliations:** 1 Department of Natural Resources TERI School of Advanced Studies New Delhi India; 2 Kumaun University Nainital India; 3 Institute of Public Health College of Medicine and Health Sciences United Arab Emirates University Al Ain United Arab Emirates; 4 Institute of Medicine Tribhuvan University Kathmandu Nepal; 5 Public Health and Infection Research Group Faculty of Health Sciences Universidad Tecnologica de Pereira Colombia, SC United States; 6 Department of Science & Technology Haryana Space Applications Centre Hisar India; 7 Anchor Systems Corporation Reston, VA United States; 8 Hughes Systique Corporation Germantown, MD United States; 9 Molecular Diagnostic Laboratory Johns Hopkins Aramco Healthcare Dhahran Saudi Arabia; 10 Department of Community Nutrition National Nutrition and Food Technology Research Institute Shahid Beheshti University of Medical Sciences Tehran Iran; 11 College of Horticulture and Forestry Rani Lakshmi Bai Central Agricultural University Jhansi India

**Keywords:** SARS-COV2, COVID-19, coronavirus, forecast, prediction, ARIMA models

## Abstract

**Background:**

The coronavirus disease (COVID-19) pandemic has affected more than 200 countries and has infected more than 2,800,000 people as of April 24, 2020. It was first identified in Wuhan City in China in December 2019.

**Objective:**

The aim of this study is to identify the top 15 countries with spatial mapping of the confirmed cases. A comparison was done between the identified top 15 countries for confirmed cases, deaths, and recoveries, and an advanced autoregressive integrated moving average (ARIMA) model was used for predicting the COVID-19 disease spread trajectories for the next 2 months.

**Methods:**

The comparison of recent cumulative and predicted cases was done for the top 15 countries with confirmed cases, deaths, and recoveries from COVID-19. The spatial map is useful to identify the intensity of COVID-19 infections in the top 15 countries and the continents. The recent reported data for confirmed cases, deaths, and recoveries for the last 3 months was represented and compared between the top 15 infected countries. The advanced ARIMA model was used for predicting future data based on time series data. The ARIMA model provides a weight to past values and error values to correct the model prediction, so it is better than other basic regression and exponential methods. The comparison of recent cumulative and predicted cases was done for the top 15 countries with confirmed cases, deaths, and recoveries from COVID-19.

**Results:**

The top 15 countries with a high number of confirmed cases were stratified to include the data in a mathematical model. The identified top 15 countries with cumulative cases, deaths, and recoveries from COVID-19 were compared. The United States, the United Kingdom, Turkey, China, and Russia saw a relatively fast spread of the disease. There was a fast recovery ratio in China, Switzerland, Germany, Iran, and Brazil, and a slow recovery ratio in the United States, the United Kingdom, the Netherlands, Russia, and Italy. There was a high death rate ratio in Italy and the United Kingdom and a lower death rate ratio in Russia, Turkey, China, and the United States. The ARIMA model was used to predict estimated confirmed cases, deaths, and recoveries for the top 15 countries from April 24 to July 7, 2020. Its value is represented with 95%, 80%, and 70% confidence interval values. The validation of the ARIMA model was done using the Akaike information criterion value; its values were about 20, 14, and 16 for cumulative confirmed cases, deaths, and recoveries of COVID-19, respectively, which represents acceptable results.

**Conclusions:**

The observed predicted values showed that the confirmed cases, deaths, and recoveries will double in all the observed countries except China, Switzerland, and Germany. It was also observed that the death and recovery rates were rose faster when compared to confirmed cases over the next 2 months. The associated mortality rate will be much higher in the United States, Spain, and Italy followed by France, Germany, and the United Kingdom. The forecast analysis of the COVID-19 dynamics showed a different angle for the whole world, and it looks scarier than imagined, but recovery numbers start looking promising by July 7, 2020.

## Introduction

### Background

At the World Health International Conference in Geneva in January 2020, the World Health Organization (WHO) announced an outbreak of the new coronavirus. The novel coronavirus (severe acute respiratory syndrome [SARS] coronavirus 2) from Wuhan, China has continued to spread around the world since January 2020 and has turned into a pandemic of the coronavirus disease (COVID-19) [1,2]. Due to the rapid spreading potential and the absence of vaccines and drugs, the contagious COVID-19 devastated normal life around the world. Currently, COVID-19 has infected more than half a million of the population, has killed more than 25,000 people, and has forced more than 3 billion to stay in their homes [3]. Many people started getting pneumonia without any reason, and most of the cases were linked to Wuhan Seafood Market, where they sell fish and trade live animals. The new coronaviruses lurking around the world are threatening our rule, and the prevalence of fear and panic is increasing. This has also affected the cryptocurrency market [4,5]. The country in which the coronavirus has caused the most devastation after China is Italy. In Italy, hundreds of people are dying every day due to this deadly virus. The corona virus is 900 times smaller than a human hair. Despite its size, this small virus has scared the whole world. In December 2019, the first case of COVID-19 came from Wuhan City in China [6-8].

During the Chinese New Year migration, the virus spread to other Chinese provinces in early and mid-January 2020. The WHO [3] revealed that cases began to be detected in other countries by international travelers. Due to a lack of knowledge about this virus, the COVID-19 pandemic placed tremendous strain on everyone around the world. To prevent further transmission, strong preventive measures have intensified week-to-week; however, the numbers of infected cases are consistently increasing around the world, even after undergoing lockdown. Mathematical approaches have been widely used to infer critical epidemiological transitions and parameters of COVID-19. Epidemic curve fitting, surveillance data during the early transmission, and other epidemic models have been frequently applied to generate forecasts of the COVID-19 pandemic across the world [9-11]. This study aims to identify the top 15 countries with the most confirmed cases with spatial mapping. A comparison was done between the identified top 15 countries for confirmed cases, deaths, and recoveries, and an advanced autoregressive integrated moving average (ARIMA) model was used for predicting the spread of COVID-19 trajectories for the next 2 months (until July 7, 2020).

### Study Area

Various studies have been presented for forecasting many epidemic diseases. This research study analyzes dynamic models to generate 20-day forecasts of cumulative confirmed deaths and recoveries from COVID-19 cases by country, territory, or conveyance generated on April 24, 2020. The United States, Spain, Italy, France, Germany, the United Kingdom, Turkey, Iran, China, Russia, Brazil, Canada, Belgium, the Netherlands, and Switzerland were taken from the top 20 countries based on cumulative effect data. The ARIMA model assigns a weight to the considered past values and an error value to correct the modelling; other basic regression and exponential models use all past values to predict future values, so the ARIMA model is preferred. This study analyzed and extracted worldwide data based upon a time series data-based advanced prediction ARIMA model approach for the top 15 COVID-19-infected countries.

## Methods

### Data

We used data from Worldometer, which reports the approximate data of cumulative cases for more than 170 countries worldwide including state- or province-level cases for some countries [12]. We have collected the case data for each day at given stipulated times, from January 21, 2020, to April 24, 2020. Furthermore, we preprocessed the top 15 countries’ data with their spatial locations to collect and create some spatial attributes for the overall available data sets to forecast the trajectory of COVID-19 cases. In addition, as whole worldwide data is not available for stipulated times, we did not create any worldwide pandemic forecasting. Some dates with cases of confirmed COVID-19 along with total cumulative results of recovered cases and death cases were analyzed using statistical analysis along with spatial extinct. We used the ARIMA model with R (R Foundation for Statistical Computing) and validated it using Akaike information criterion (AIC). The new projected data was used up to July 2, 2020, for the creation of a trajectory projected score for each category: case confirmed, recovered, and death.

Recent reported cumulative data of confirmed cases, deaths, and recoveries of COVID-19 from January 21 to April 26, 2020, were obtained from Worldometer. The reported data were used to predict more than 60 days and to understand the positive effects in the near future as well as the projected trends over trajectories. The different statistical phenomenological models in the R-language platform were used to analyze the disease-based trajectories model for prediction purposes. The four models were used to analyze the aggregate data set for time series analysis. This includes the ARIMA model, which is a mass model of two different models, including the autoregression (AR) model and the moving average (MA) model [13]. This model also used AIC statistics and coverage of regression analysis.

Another type of COVID-19, like SARS disease, was analyzed without breaking the current situation or predicting the future perspective [14]. The vector auto-average model was used to predict the spatial extinct while using remote sensing data for the purpose of the creation of a worldwide geographic information system (GIS) map for three different variables [15]. These three variables in the GIS environment created a map of cumulative confirmed cases by country as well as recovered and death maps [16]. The use of another statistical analysis was a generalized logistic growth model, which generally is depicted as a scaling parameter for integrating an additional result-oriented value put method [17]. Some epidemic models used in disease epidemic conditions measure oscillates, which are multiple peak parameters inferred in subepidemic and pandemic conditions to determine the projected outcomes [18].

After standardizing all the models, the data of the top 20 countries were included to analyze the forecasting models of differential spatial adjacent and projected trajectories, which were analyzed up to July 2, 2020. We used the GIS and remote sensing to determine the pandemic mapping and analyze the upcoming effects of COVID-19.

### ARIMA

MA is the present value of a series, which is defined as a linear combination of past errors. Assuming the errors to be independently distributed with the normal distribution [13,19], order q is defined as:

y_t_ = c + ε_t_ + θ_1_y_t–1_ + θ_2_y_t–2_ + ….….….….… + θ_q_y_t–q_**(1)**

Where:

- ε_t_=white noise
- y_t-1_ and y_t-2_=lags

Order q of the MA process is obtained from the autocorrelation function (ACF) plot; this is the lag after which ACF crosses the upper confidence interval for the first time. We combined differencing with MA and AR models, and the combined model can be expressed as:

y′_t_ = c + ϕ_1_y′_t–1_ + ϕ_2_y′_t–2_ + ... + ϕ_p_y′_t–p_ + θ_1_y_t–1_ + θ_2_y_t-2_ + ….… + θ_q_y_t–q_ + ε_t_**(2)**

Here, y′_t_ is the differenced series. The “predictors” on the right-hand side include both lagged values of y_t_ and lagged errors. We call this an ARIMA (p, d, q) model, where:

- q=order of the MA part
- d=degree of first differencing involved
- p=order of the AR part

## Results

**Figure 1:**
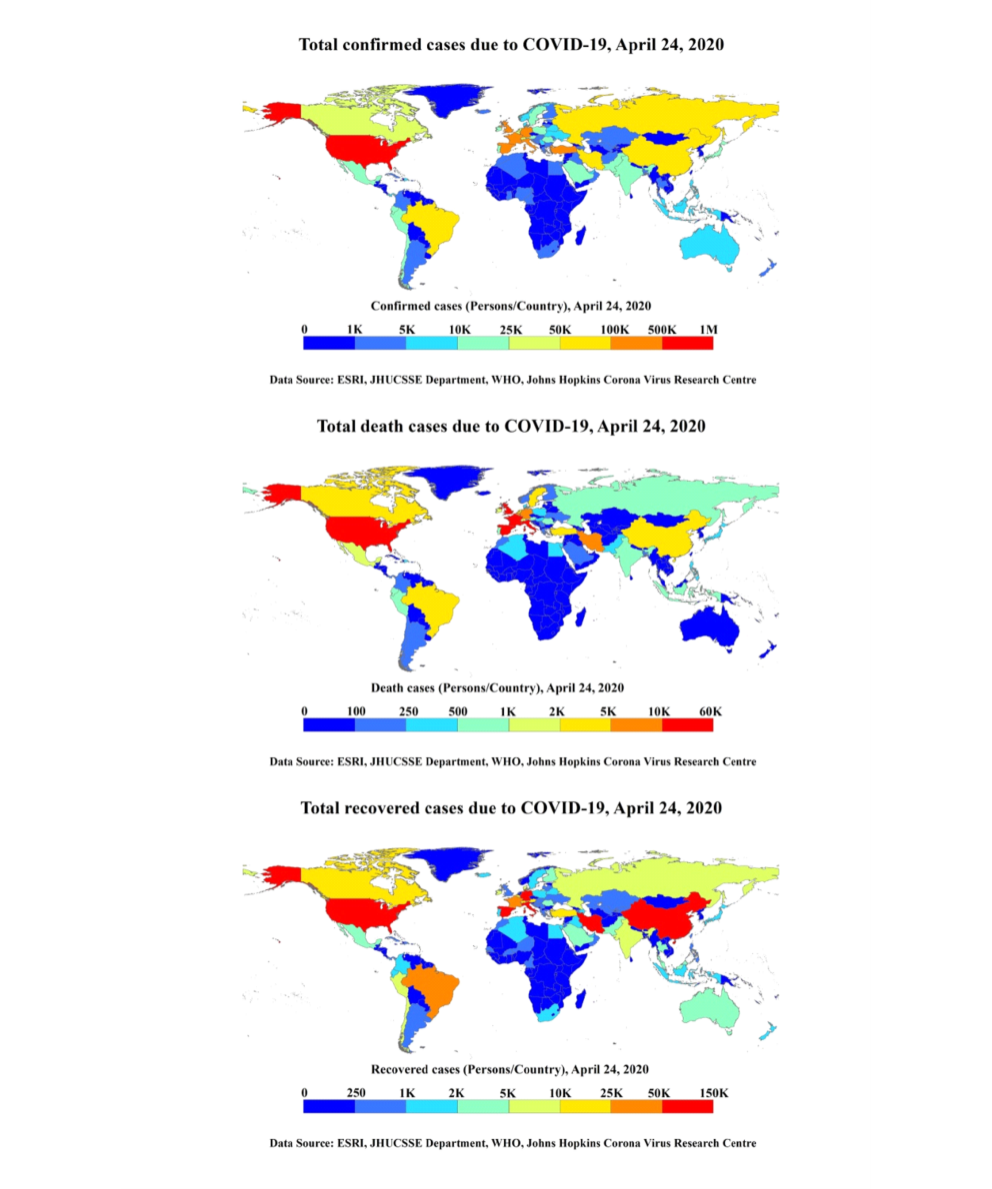
COVID-19 pandemic spatial pattern of total confirmed cases (top), deaths (middle), and recoveries (bottom) from January 19 to April 24, 2020, in countries and territories. COVID-19: coronavirus disease; JHUCSSE: Johns Hopkins University Center for Systems Science and Engineering; WHO: World Health Organization.

**Figure 2:**
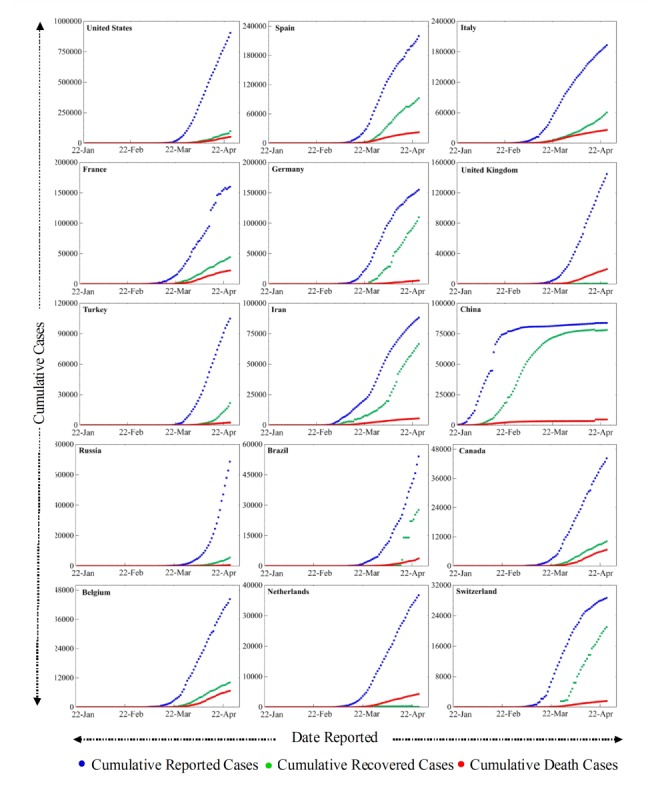
Comparisons between cumulative confirmed cases, recoveries, and deaths of coronavirus disease in the top 15 affected countries.

The top 15 countries were identified using mapping of cumulative confirmed COVID-19 cases from January to April 24, 2020, for 200 nations as presented in Figure 1. The top 15 countries with a high number of confirmed cases were stratified to include the data in a mathematical model. The top 15 countries’ (the United States, Spain, Italy, France, Germany, the United Kingdom, Turkey, Iran, China, Russia, Brazil, Canada, Belgium, the Netherlands, and Switzerland) cumulative cases, deaths, and recoveries from COVID-19 were compared in Figure 2. The United States, The United Kingdom, Turkey, China, and Russia saw a relatively fast spread of the disease. There was a fast recovery ratio in China, Switzerland, Germany, Iran, and Brazil, but a slow recovery ratio in the United States, the United Kingdom, the Netherlands, Russia, and Italy as shown in Figure 2. In addition, there were higher death rate ratios in Italy and the United Kingdom, and lower death rate ratios in Russia, Turkey, China, and the United States (Figure 2).

**Figure 3:**
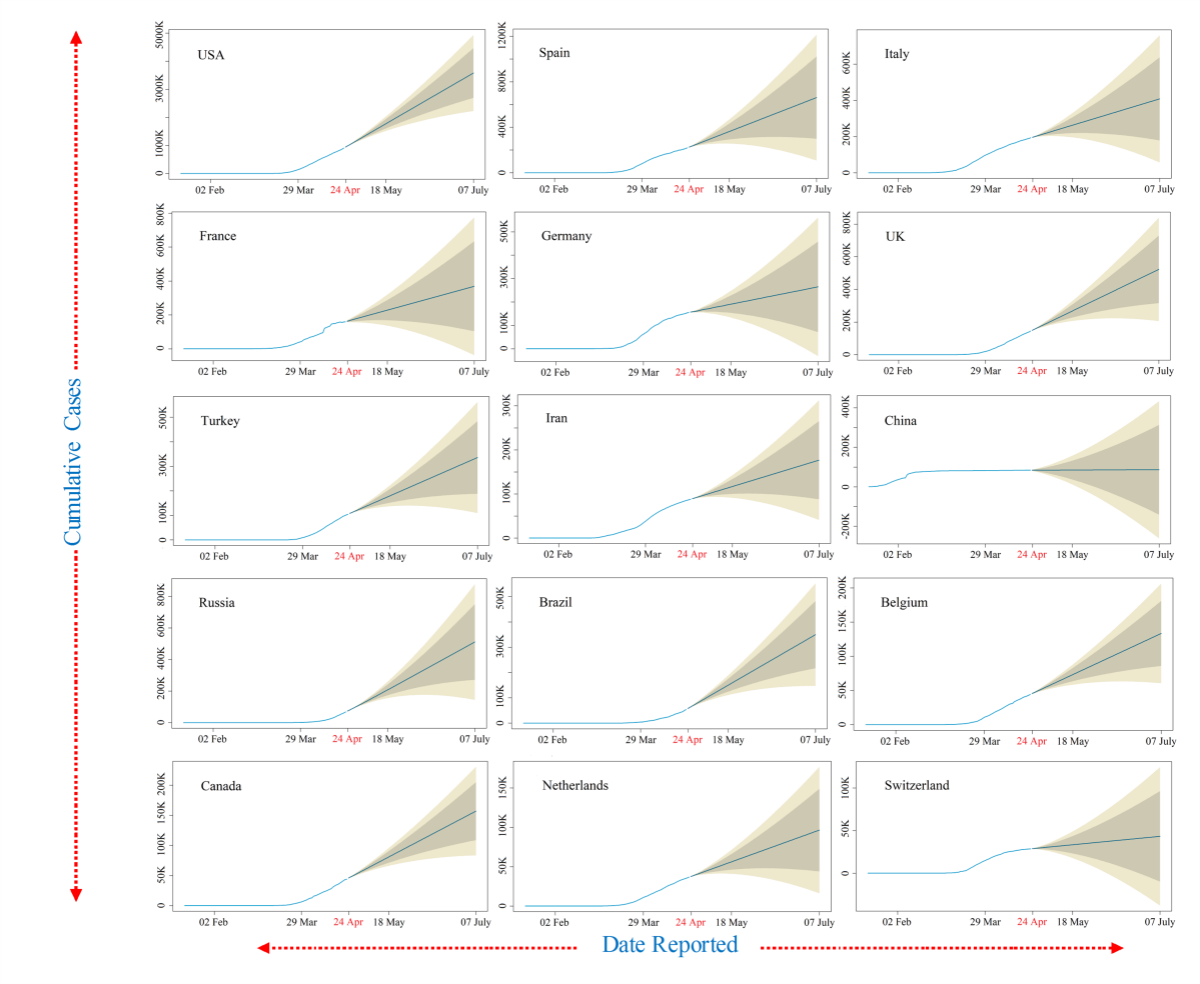
The autoregressive integrated moving average model prediction for more than 2 months of cumulative confirmed coronavirus disease cases in the top 15 affected countries shown in a cyan color (95% confidence).

**Figure 4:**
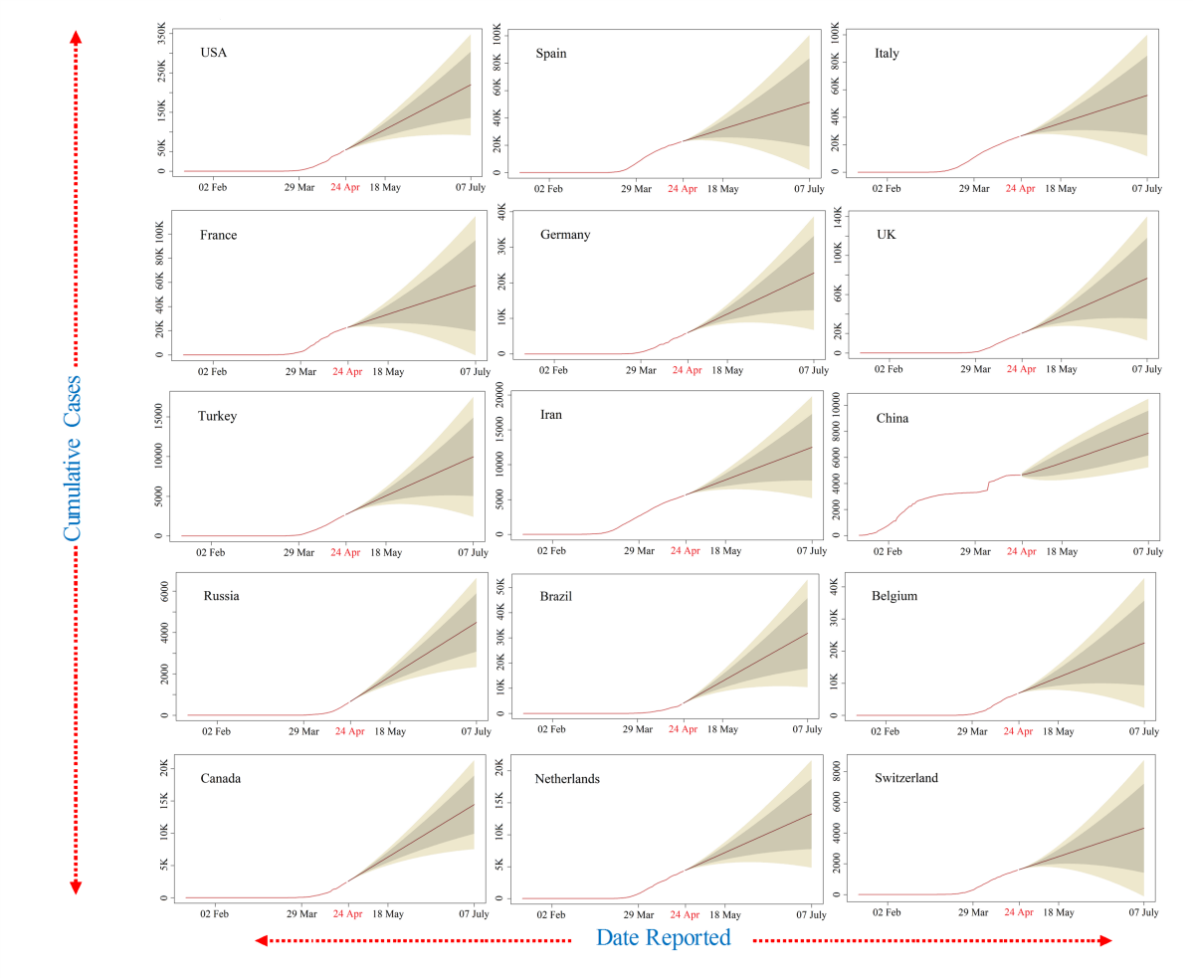
The autoregressive integrated moving average model prediction for more than 2 months of cumulative confirmed coronavirus disease cases in the top 15 affected countries shown in a brown color (95% confidence).

**Figure 5:**
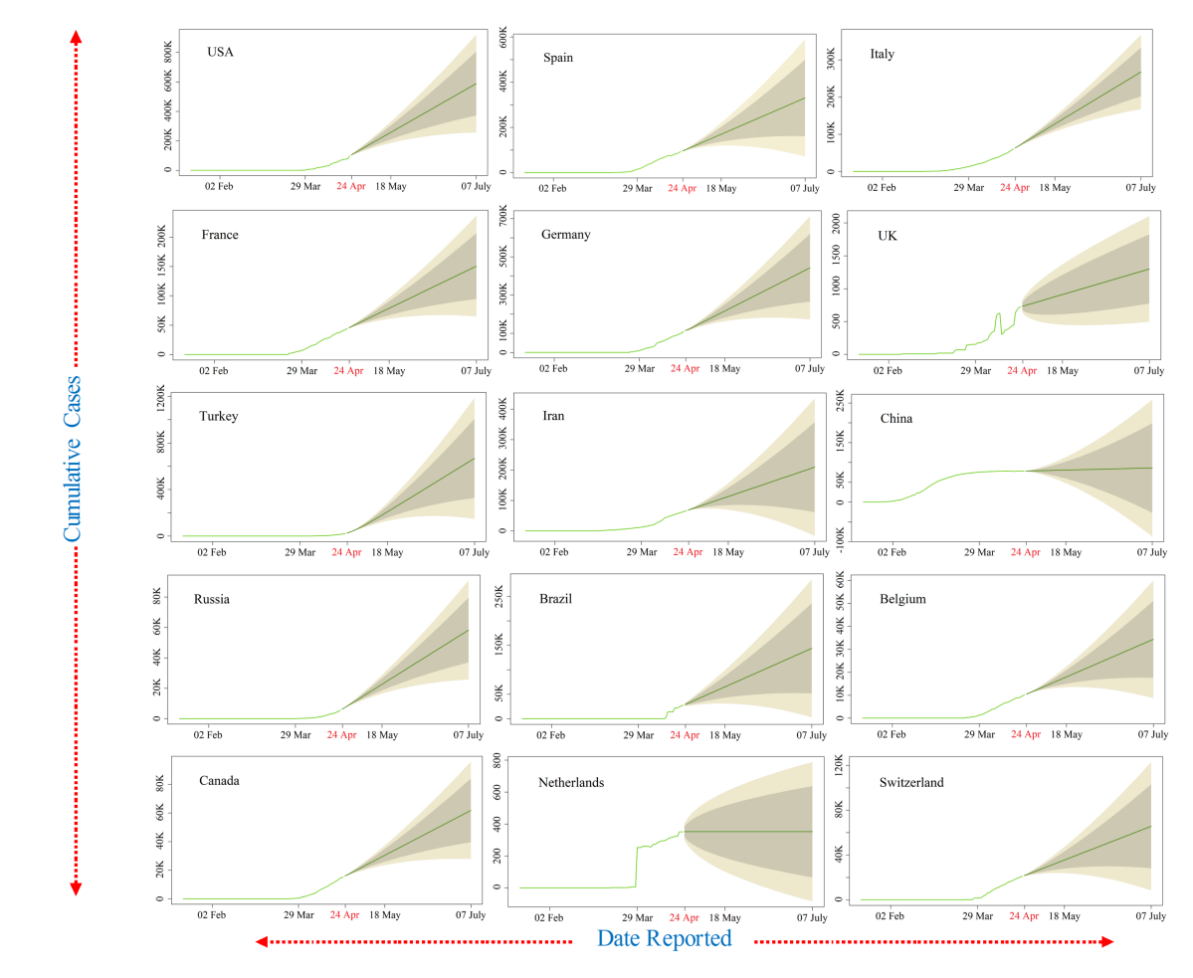
The autoregressive integrated moving average model prediction for more than 2 months of cumulative confirmed coronavirus disease cases in the top 15 affected countries shown in a green color (95% confidence).

Furthermore, data smoothening was applied to stabilize the data by removing changes in the level of a time series and, therefore, eliminating (or reducing) the trend and seasonality. After this, the forecast prediction model was applied by using AR and MA models to generate plots of the different trends in upcoming days. The ARIMA model was validated for the available current data using the AIC value; it estimates that the out-of-sample prediction error and lowest value are preferable. Its value were around 20, 14, and 16 for cumulative confirmed cases, deaths, and recoveries from COVID-19, respectively, which represents less error. The outcome of these predictions is presented in Figure 3. Our findings revealed linearity in the confirmed cumulative cases and showed a rapid exponential growth phase in the world, which might occur roughly from April 8 to April 24, 2020, when the number of COVID-19 cases may rise steeply to nearly 1 million in the United States, 220,000 in Spain, 200,000 in Italy, 180,000 in France, and 190,000 in Germany. Other countries that have a smaller number of cases but show a declining upward trend include Switzerland, Germany, and Italy (Figure 2). However, the cases of COVID-19 in China remain stable (Figure 2). The ARIMA model predicted confirmed cases, deaths, and recoveries for the next month from April 24 to July 7, 2020, using the past 3 months of data in Figure 3 (cyan color), Figure 4 (brown color), and Figure 5 (green color) with 95% confidence intervals. Along with the 95% confidence predicted line after April 24, the 80% and 70% confidence wide values are shown in light grey and light-yellow colors, respectively. The wide confidence intervals help to manage any sudden changes in the prediction of dynamic COVID-19 cases.

During the next 2 months between April 24 and July 7, 2020, the model predicted that the confirmed cases, deaths, and recoveries would be doubled in all countries except China, Switzerland, and Germany (Figures 3-5). It was also observed that the death and recovery rates will be faster when compared to confirmed cases during the next 2 months. The associated mortality rate will be much higher in the United States, Spain, and Italy followed by France, Germany, and the United Kingdom. The recovery rates will stay slow at first but then rapidly increase in the United States, Italy, Germany, and France by the end of June 2020 (Figure 5).

## Discussion

### Principal Findings

The COVID-19 daily data was collected and cumulatively represented as a spatial map for more than 170 countries and territories. The spatial map is useful to identify the intensity of COVID-19 infections in the top 15 countries and the continents. The recent reported data for confirmed cases, deaths, and recoveries for the last 3 months from January to April 2020 was represented and compared between the top 15 infected countries. The ARIMA model was used to predict estimated confirmed cases, deaths, and recoveries for the top 15 countries from April 24 to July 7, 2020. Its value was represented with 95%, 80%, and 70% confidence intervals, and the 95% confidence intervals were shown as the median interval between the 80% and 70% wide values. The validation of the ARIMA model was carried out using the AIC for the available recent data; its values were about 20, 14, and 16 for cumulative confirmed cases, deaths, and recoveries from COVID-19, respectively, which represents acceptable results. The observed predicted values showed that the confirmed cases, deaths, and recoveries will double in all countries except China, Switzerland, and Germany. It was also observed that the death and recovery rates were faster when compared to confirmed cases during the next 2 months. The associated mortality rate will be much higher in the United States, Spain, and Italy followed by France, Germany, and the United Kingdom. The limitation of the ARIMA model is that it does not support any volatility or in-between changes in the prediction periods. The accuracy of the countries’ data accumulated from Worldometer was a matter of trust for the representation of the whole study.

The forecast analysis of COVID-19 dynamics showed a different angle for the whole world, and it looks scarier than imagined. Interestingly, the recovery numbers also look promising, with resistance starting by July 2020. Thus, a slowdown in the surge of the COVID-19 pandemic during the proceeding months depends upon various administrative interventions and public awareness about the spread of the COVID-19 pandemic.

## Abbreviations

ACF: autocorrelation function
AIC: Akaike information criterion
AR: autoregression
ARIMA: autoregressive integrated moving average
COVID-19: coronavirus disease
GIS: geographic information system
MA: moving average
SARS: severe acute respiratory syndrome
WHO: World Health Organization

## References

[1] Zou L, Ruan F, Huang M, Liang L, Huang H, Hong Z, Yu J, Kang M, Song Y, Xia J, Guo Q, Song T, He J, Yen H, Peiris M, Wu J (2020). SARS-CoV-2 viral load in upper respiratory specimens of infected patients. N Engl J Med.

[2] Xu X, Wu X, Jiang X, Xu K, Ying L, Ma C, Li S, Wang H, Zhang S, Gao H, Sheng J, Cai H-L, Qiu Y-Q, Li L-J (2020). Clinical findings in a group of patients infected with the 2019 novel coronavirus (SARS-Cov-2) outside of Wuhan, China: retrospective case series. BMJ.

[3] World Health Organization.

[4] Kuniya T (2020). Prediction of the epidemic peak of coronavirus disease in Japan, 2020. J Clin Med.

[5] Linton NM, Kobayashi T, Yang Y, Hayashi K, Akhmetzhanov AR, Jung S, Yuan B, Kinoshita R, Nishiura H (2020). Incubation period and other epidemiological characteristics of 2019 novel coronavirus infections with right truncation: a statistical analysis of publicly available case data. J Clin Med.

[6] European Centre for Disease Prevention and Control.

[7] Jung S-M, Akhmetzhanov AR, Hayashi K, Linton NM, Yang Y, Yuan B, Kobayashi T, Kinoshita R, Nishiura H (2020). Real time estimation of the risk of death from novel coronavirus (2019-nCoV) infection: inference using exported cases. medRxiv.

[8] Nishiura H, Kobayashi T, Yang Y, Hayashi K, Miyama T, Kinoshita R, Linton NM, Jung S, Yuan B, Suzuki A, Akhmetzhanov AR (2020). The rate of underascertainment of novel coronavirus (2019-nCoV) infection: estimation using Japanese passengers data on evacuation flights. J Clin Med.

[9] Kucharski AJ, Russell TW, Diamond C, Liu Y, Edmunds J, Funk S, Eggo RM, Sun F, Jit M, Munday JD, Davies N, Gimma A, van Zandvoort K, Gibbs H, Hellewell J, Jarvis CI, Clifford S, Quilty BJ, Bosse NI, Abbott S, Klepac P, Flasche S (2020). Early dynamics of transmission and control of COVID-19: a mathematical modelling study. Lancet Infect Dis.

[10] Liu Y, Gayle A, Wilder-Smith A, Rocklöv J (2020). The reproductive number of COVID-19 is higher compared to SARS coronavirus. J Travel Med.

[11] Pirouz B, Shaffiee Haghshenas S, Shaffiee Haghshenas S, Piro P (2020). Investigating a serious challenge in the sustainable development process: analysis of confirmed cases of COVID-19 (new type of coronavirus) through a binary classification using artificial intelligence and regression analysis. Sustainability.

[12] (2020). Worldometer.

[13] Chowell G (2017). Fitting dynamic models to epidemic outbreaks with quantified uncertainty: a primer for parameter uncertainty, identifiability, and forecasts. Infect Dis Model.

[14] Roosa K, Chowell G (2019). Assessing parameter identifiability in compartmental dynamic models using a computational approach: application to infectious disease transmission models. Theor Biol Med Model.

[15] Wang X, Wu J, Yang Y (2012). Richards model revisited: validation by and application to infection dynamics. J Theor Biol.

[16] Richards FJ (1959). A flexible growth function for empirical use. J Exp Bot.

[17] Roosa K, Lee Y, Luo R, Kirpich A, Rothenberg R, Hyman J, Yan P, Chowell G (2020). Real-time forecasts of the COVID-19 epidemic in China from February 5th to February 24th, 2020. Infect Dis Model.

[18] Chowell G, Tariq A, Hyman JM (2019). A novel sub-epidemic modeling framework for short-term forecasting epidemic waves. BMC Med.

[19] Beets P, Kimberley M, Oliver G, Pearce S (2014). The application of stem analysis methods to estimate carbon sequestration in arboreal shrubs from a single measurement of field plots. Forests.

